# Multiannual, Intensive Strength-Endurance Training Modulates the Activity of the Cardiovascular and Autonomic Nervous System among Rowers of the International Level

**DOI:** 10.1155/2019/3989304

**Published:** 2019-10-02

**Authors:** Tomasz Kowalik, Jacek J. Klawe, Małgorzata Tafil-Klawe, Witold Słomko, Joanna Słomko, Anna Srokowska, Andrzej Lewandowski, Paweł Zalewski

**Affiliations:** ^1^Institute of Physical Education, Kazimierz Wielki University, Bydgoszcz, 2 85-091, Poland; ^2^Department of Hygiene, Epidemiology and Ergonomics, Nicolaus Copernicus University, Karlowicza 24, Bydgoszcz 85-092, Toruń, Poland; ^3^Department of Human Physiology, Nicolaus Copernicus University, Karlowicza 24, Bydgoszcz 85-092,, Toruń, Poland; ^4^Department of Physiotherapy, Nicolaus Copernicus University, Bydgoszcz 3 85-801, Toruń, Poland; ^5^Department of Fundamentals of Physical Culture, Nicolaus Copernicus University, ul. Swietojanska 20, Bydgoszcz 85-094, Toruń, Poland

## Abstract

**Introduction:**

Professional athlete training is significantly different from recreational physical activity, and sustained, repetitive exposure to over-strenuous and intensive training may result in critical changes of most systems and organs in a sportsman's body.

**Aim:**

The assessment of the influence of multiannual strength-endurance training on the autonomic nervous system (ANS) and cardiovascular system (CVS) among the rowers of Polish national team.

**Materials and Methods:**

20 rowers, aged 20–30, seniors of Polish national team were qualified into the study. The functional examination of ANS was conducted by means of Task Force® Monitor system. The assessed parameters included hemodynamic parameters, heart rate, and blood pressure variability and reflexes sensitivity of baroreceptors. In order to examine and compare the reaction of autonomic nervous system the subjects underwent a tilt test.

**Results:**

In the study group, significantly higher levels of sBP (129.3  ±  12.2 vs 118.3 ± 8.4, *p* = 0.0030), SI (59.9 ± 8.8 vs 41.2 ± 6.8, *p* > 0.001), CI (3.2 ± 0.5 vs 2.4 ± 0.4, *p* > 0.001), and significantly lower levels of HR (54.2 ± 5.3 vs 60.1 ± 5.7, *p* = 0.0034) and TPRI (2333.3 ± 389.9 vs 2950.2 ± 604.2, *p* = 0.0012) compared to the control group, were found. After the tilt test the levels of HR (*p* = 0.0005) and TPRI (*p* = 0.0128) were significantly higher but SI (*p* > 0.001) and CI (*p* = 0.0006) were significantly lower in the study group compared to the control.

**Conclusions:**

Multiannual strength-endurance training connected with rowing activities substantially modulates the activity of cardiovascular and autonomic nervous system, influences the volumetric workload of the heart and structural changes, and increases the sensitivity of reflexes of arterial baroreceptors.

## 1. Introduction

Active lifestyle and regular endurance (oxygen) trainings have a positive influence on the cardiac muscle and other components of the cardiovascular system [1]. However, it is not always true in case of professional sport training because the main aim of such training is to increase the performance of the body—very often by all means. Due to that, professional athlete training is significantly different from recreational physical activity and sustained, repetitive exposure to over-strenuous and intensive training may result in critical changes of most systems and organs in a sportsman's body [2–5]. Professional sport training requires considerable adaptive changes in a rower's body. Changes induced by strength-endurance training affect mainly respiratory and cardiovascular systems. Rowing combines the strength and endurance effort. This is why rowers may suffer from concentric and eccentric myocardial wall hypertrophy and the extension of cardiac cavities. As a result, rowers' hearts may reach record sizes e.g., heart mass exceeding 500 g and stroke volume reaching 190 ml [6]. What is more, adaptive changes in the cardiovascular system include the changes dependent on nervous regulations as well as metabolic and morphological changes. The research conducted on rowers [7, 8], runners [9] and cross-country skiers [10–13] showed that moderate endurance training positively influences autonomic nervous system (ANS) causing the growth of parasympathetic activity and the reduction of sympathetic nervous system agitation. The available literature data lack reliable sources which describe the influence of strength-endurance training on the activity of autonomic nervous system in terms of regulatory mechanisms of cardiovascular system associated with the assessment of the heart muscle inotropy among sportsmen with an increased level of training. Due to that, the aim of this paper was the assessment of hemodynamic parameters, cardiac contractility, work of right and left ventricles and functional parameters of autonomic nervous system among rowers of international master class at a rest period.

## 2. Materials and Methods

### 2.1. Test Group

The research was conducted on 20 rowers of Polish senior national team at a rest period after a starting season. The criteria for inclusion in the research included: sex, professional rowing practice (at least 10 years), good general health condition, and lack of any cardiovascular and autonomic nervous systems diseases. In order to maintain homogeneity, the clinical reference group included 20 men aged 25–35. All men were professionally active and were characterised by general good health condition, lack of ANS diseases, and healthy cardiovascular system. The characteristics of the basic biological traits of the subjects are shown in Table 1.

### 2.2. Training Characteristics

The examination was conducted in a training microcycle in a resting phase. The period lasted 4–6 weeks and was characterised by gradual decrease in starting performance—after the end of the starting season. During that period, the trainings were dominated by general physical exercises: running, gym exercises, gymnastics, team sports, or swimming. In order to measure the physical endurance, the rowers underwent the test of gradually growing strength on Concept II rowing machine. The test included eight three-minute stages, separated by thirty-second breaks. The load in the test started from 170 W and was increased with each stage by 50 W at the rowing pace starting from 14 strokes/minute and increased by 2 strokes/minute in each stage. During the thirty-second breaks a blood sample was taken from an earlobe with Super GL 2 device (dr Müller, Germany) in order to mark the lactate concentration. The heart beat frequency (HR) was persistently monitored with a Sport Tester (Polar) and indicators of gas exchange were tested with a mobile MetaMax 3B device (Cortex, Germany). The average value of VO_2max_ among rowers was 6.17 l/min with the average threshold value (4 mmol) which equals 378.75 W. The maximum average acidification ability was 14.10 mmol/l with the maximum ultimate power 496 W.

### 2.3. Examination Protocol

The test took place in the morning, 30 minutes after a meal. In order to eliminate the impact of stress related to participation in the research, each subject was thoroughly informed and acquainted with the methodology of the research and methods applied. All examinations were conducted in the conditions complying with the criteria of functional examinations of the autonomic nervous system. The examination room was deafened and darkened, and the air conditioning system kept the temperature and humidity constant. The functional examination of the autonomic nervous system was a non-invasive procedure thanks to Task Force® Monitor system. The assessed parameters included hemodynamic parameters, cardiac contractility, work of left and right ventricles, heart rate, blood pressure variability, and sensitivity of reflexes of baroreceptors.

### 2.4. The Assessment of Hemodynamic Parameters, Cardiac Contractility, and the Work of Left and Right Ventricles

The recording of the biological signals was non-invasive thanks to Task Force® Monitor system (3040i model, CNSystems) and subject-friendly equipment used. Task Force Monitor**®**measuring system includes the following components: (1) a device for a continuous measurement of blood pressure (contBP), (2) two-channel electrocardiograph (ECG), (3) impedance cardiograph (ICG), and (4) a device for oscillometric measurement of blood pressure (oscBP). All the components are operated by means of the integrated computer system and all, except for the oscBP device, record biological signals in a *beat-to-beat *mode. The system calibrates automatically thanks to its integrated generators. Measurements which are false or exceed the biological norms are detected and automatically deleted by the TFM system [14, 15].

### 2.5. The Spectral Analysis of the Heart Rate and Blood Pressure Variability (HRV and BPV)

In order to assess the changes in autonomic nervous system an indirect method—spectral analysis—was used. It is based on the analysis of RR interval and the signal of blood pressure in a continuous mode i.e., heart beat by heart beat. The signals are transformed from the time domain into the frequency domain [18–20]. The calculations of the spectrum power are done by means of the advanced mathematical functions, Task Force Monitor system uses the algorithm of the adaptive autoregressive parameter (ARR).

### 2.6. The Parameters of Sensitivity of the Reflexes of Arterial Baroreceptors

Spontaneous activity of the reflexes of arterial baroreceptors was measured using a sequence method. The method is based on establishing at least three subsequent heart beats during which there is an observed increase in systolic blood pressure and extension of RR interval—a cumulative sequence, or a decrease in systolic blood pressure and shortening of RR interval—a decreasing sequence. The thresholds are calculated with the accuracy of 1 mmHg for the systolic blood pressure and 6 ms for the RR interval [15, 16, 17].

### 2.7. Statistical Analysis

Standard of continuous variables distribution was formulated with the Shapiro-Wilk test, and the statistical characteristics were shown as arithmetic means and standard deviations (±SD). In order to assess the significance of the variations between the measured values obtained in the group of rowers and in the clinical control group, two tests—Student's *t*-test and Mann–Whitney *U* test—were used depending on the distribution characteristics of the analysed variables. All the calculations were made using *Statistica 9.0* software (StatSoft, USA) where the *p* ≤ 0.05 value was considered statistically significant.

## 3. Results

### 3.1. Resting Position

#### 3.1.1. Hemodynamic Parameters

The rowers scored significantly higher on systolic blood pressure (sBP), stroke index (SI), cardiac index (CI) and all sequences of baroreceptors (BRS), as well as substantially lower on heart rate (HR), and total peripheral resistance index (TPRI) (Table 2).

#### 3.1.2. Contractility and Ventricular Functions Parameters

It was observed that the rowers obtained higher results of index of contractility (IC), acceleration index (ACI), Heather index (HI) and left ventricular work index (LVWI) and substantially lower results of systolic time ratio (STR) than subjects in the control group (Table 3).

#### 3.1.3. Spectral Analysis of Heart Rate Variability (HRV)

In comparison to the control group, in a resting position, the rowers scored significantly higher on the low spectral component values (LF-RRI, *p* = 0.0269) and higher on the frequency of the HRV spectrum (HF-RRI, *p* = 0.0148) as well as spectral HRV power density (PSD-RRI, *p* = 0.0248) (Table 4).

### 3.2. Responses to the Tilt Test

After the tilt test, the rowers, in comparison to the control group, obtained much higher results of heart rate (HR, *p* = 0.0005) and total peripheral resistance index (TPRI, *p* = 0.0128) as well as lower results of stroke index (SI, *p* > 0.001) and cardiac index (CI, *p* = 0.0006). The groups did not differ significantly in systolic blood pressure (sBP, *p* = 0.6119), diastolic blood pressure (dBP, *p* = 0.0671), and mean blood pressure levels (mBP, *p* = 0.3424) (Table 5).

## 4. Discussion

Multiannual strength-endurance rowing training substantially decreases heart rate and modulates the function of autonomic nervous system. One of the main results of professional rowing training is the increase volumetric workload of the heart and structural changes related to that.

The examined rowers had significantly higher average values of stroke volume (SV) and stroke index (SI) than men in the reference group, as well as substantially higher level of cardiac output (CO) and its derivative—cardiac index (CI). It is worth noticing that the abovementioned changes occurred despite significantly lower heart rate (HR) compared to the reference group. The only significant differences between the two groups in terms of resting parameters of blood pressure concerned systolic blood pressure (sBP), which was significantly higher in the rowers than reference group. The results show a possible increase in cardiac output and stroke volume and a significant decrease in the average value of vascular resistance and total peripheral resistance index in the group of rowers, which are probably the result of better development of muscular layer of arterial walls. The results prove that rowing-related effort is mainly of dynamic nature. Similar adaptive changes of cardiovascular system to the sustained and regular physical effort were shown in the recently published research of men aged >60 who used to be professional athletes and were physically active throughout their lives [18]. An increase in cardiac output has been observed as a result of not only a dynamic training but also among people exposed to a sustained endurance training. Several papers show that cardiac output of sportsmen who practice endurance disciplines may reach 5–6 l/min at rest and over 40 l/min during maximum effort [17, 18]. Endurance training is connected with the increase in volumetric workload of the heart, a significant extension of the left ventricle and a slight thickening of its walls. In the literature these changes are referred to as “sportsmen heart” [18]. In order to examine more thoroughly the influence of strength-endurance training of rowers on the heart muscle, and myocardium contractility parameters were measured. The examined sportsmen, in comparison to the reference group, scored substantially higher in the average values of cardiac impedance parameters which define heart inotropy i.e., index of contractility (IC) characterised by maximum blood flow during left ventricle ejection, acceleration index (ACI) defining maximum blood flow acceleration in the aorta and Heather index which is the time of obtaining maximum contractility strength (capability of maximum ejection) by the left ventricle. Statistically relevant differences between the two groups in terms of index of contractility, acceleration index and Heather index were accompanied by changes of some spectral analysis of autonomic nervous system parameters, typical of an increased parasympathetic stimulation. Therefore, variations of cardiac contractility parameters observed in the subjects seem to be not only the result of cardiac reconstruction, but also a result of autonomic nervous system tension changes. Similar conclusions were drawn by the authors of a recently conducted comparative study of a group of professional runners and a control group of people of the same age who are not physically active [19]. The echocardiographic analysis showed that in the rest conditions and during low-intensity effort (50% VO_2max_) the compared groups differed only by morphometric characteristics of the left ventricle. The relevant differences of the left ventricle systolic activity (higher values of peak systolic velocity in the sportsmen group) were revealed only during the effort equal to 60%–100% VO_2max_ [19]. Also, a series of other examinations did not prove that sportsmen differ from people of regular level of physical activity in terms of cardiac contractility at rest [13–20]. The observations indicate that in the effort mechanism of cardiac inotropy growth, functional changes in a body play more important role than the cardiac reconstruction.

Both the literature data and the results of this study show that the main functional element significant for improvement of cardiac inotropy of professional sportsmen is the activity of autonomic nervous system. As it was previously mentioned, the study showed a few relevant differences in resting parameters of heart rate variability and blood pressure variability indexes between the rowers and the control group. But for the growth of LF-RRI, the results of HRV and BPV spectral analysis indicated a minor domination of parasympathetic system in the group of rowers. The increased parasympathetic stimulation is supported by the higher number of sequences from baroreceptors and significantly higher average slope of the function curve of linear regression of all baroreceptors registered in the sequence recorded among the rowers. In present times it is a fact that regular moderate endurance effort has a healthy influence on ANS. It lowers the sympathetic activity and increases the parasympathetic activity. These changes cause a decrease in heart rate at rest and during submaximal effort [15–17]. Recent studies on sportsmen prove that spectral analysis of heart rate and blood pressure variable can act as a measure of good training load selection.

The tilt test showed a correct response of the rowers and the reference group to an orthostatic stimulus, as shown in the growth of systolic (sBP), diastolic (dBP) and mean (mBP) blood pressure, which is the result of the containment of intracardiac parasympathetic activity, intensification of vasoconstrictive sympathetic activity and growth of total peripheral resistance (TPR). What is more, the impact of orthostatic stimulus resulted in a significant decrease in cardiac contractility parameters: index of contractility (IC) and acceleration index (ACI) along with the growth of systolic time ratio (STR). Both groups were characterised by the decrease in parasympathetic activity as shown in the decreased HR-RRI value and growth of the total number of baroreceptors sequence (*total-events event count*). All these changes reflect adaptation to the post-tilt decrease in venous return and lower level of ventricle filling. The analysed data gathered after the tilt test showed that the compared groups obtained diversified results of the response to orthostatic stimulus. In the group of rowers, it was noticed that as a result of the tilt test there was a significant decrease in stroke volume (SV), stroke index (SI), index of contractility (IC), acceleration index (ACI), parasympathetic activity (a decrease in HR-RRI and HF-dBP), and average slope of the function curve of linear regression of all baroreceptors sequences (*total-events slope mean*). What is more, the group of rowers reacted by a substantially higher growth of the heart rate (HR), total peripheral resistance (TPR), total peripheral resistance index (TPRI), and systolic time ratio (STR), as well as significantly less perspicuous growth of left ventricular work index (LVWI) and the number of total baroreceptors sequences (*total-events event count*). Rowers, contrary to the control group, showed a decrease in cardiac output (CO), cardiac index (CI), and Heather index (HI). Although most of these differences between the two groups seem to be a derivative of the discrepancies observed during the examination at the start position, some of them (e.g. growth of HP, TPR and TPRI as well as decrease in HR-RRI and HF-dBP) may also reflect a faster and more effective adaptation of sportsmen's cardiovascular systems to sudden changes of hemodynamic conditions.

There is little literature data on the influence of orthostatic stimulus on sportsmen and people who are not physically active, and the results obtained in such studies are frequently inconsistent [19]. In one of the studies, it was shown that professional sportsmen (both at the peak of their training cycle and during a postseason break) show more intense response of the sympathetic nervous system than healthy people in the control group who are not physically active [20]. Posttilt test intensification of the influence of the sympathetic nervous system on the heart rate of people who took part in a six-month aerobic exercise programme was also observed by Zhang et al. [20]. These observations seem to be partially compatible with the results obtained in the study on the rowers, in particular with the growth of heart rate, total peripheral resistance, and a decrease in the level of parasympathetic stimulation. However, it is worth noticing that in the other two studies, it was not proven that a physical training has a significant influence on the cardiovascular system during the tilt test [18–20].

## 5. Conclusions

Multiannual rowing training substantially modulates the activity of autonomic nervous system by increasing the volumetric workload of the heart and structural changes related to that, and intensifies the sensitivity of the reflexes of arterial baroreceptors, which seems to be beneficial when practising a strength-endurance sport discipline. Due to that, the observed changes, which contrast with the observation results of young healthy men, justify the necessity to monitor them in order to prevent possible pathological effects.

## Figures and Tables

**Table 1:** Basic characteristics of the subjects.

Trait	Rowers (*n* = 20)		Reference group (*n* = 20)	
	Average	SD	Average	SD
Age [years]	31.50	5.61	35	8.14
Body height [m]	1.89	0.05	1.78	0.08
Body weight [kg]	83.70	6.30	78.90	9.73
BMI [kg/m^2^]	24.67	1.15	24.40	3.11
resting sBP [mmHg]	121.28	8.27	118.31	8.44
resting dBP [mmHg]	76.84	6.54	76.51	7.26

**Table 2:** Functional parameters of cardiovascular system in resting position.

Parameter	Rowers (*n* = 20)	Reference group (*n* = 20)	*p*
HR (1/min)	54.2 ± 5.3	60.1 ± 5.7	0.0034
sBP (mmHg)	129.3 ± 12.2	118.3 ± 8.4	0.0030
dBP (mmHg)	77.7 ± 10.3	76.5 ± 7.2	0.6784
mBP (mmHg)	94.8 ± 10.8	90.85 ± 7.8	0.2096
SI (ml/m^2^)	59.9 ± 8.8	41.2 ± 6.8	*p* > 0.001
CI (l/(min^∗^m^2^))	3,2 ± 0,5	2.4 ± 0.4	*p* > 0.001
TPRI (dyn^∗^s^∗^m^2^/cm^5^)	2333.3 ± 389.9	2950.2 ± 604.2	0.0012
BRS	37.4 ± 17.6	25.2 ± 17.1	0.0438

**Table 3:** Contractility, contraction strength, and ventricular functions parameters.

Parameter	Rowers (*n* = 20)	Reference group (*n* = 20)	*p*
IC (1000/s)	67.3 ± 13.4	44.0 ± 12.2	*p* > 0.001
ACI (100/s^2^)	89.0 ± 18.5	64.6 ± 19.1	0.0004
HI (1/s^2^)	0.3 ± 0.0	0.2 ± 0.0	*p* > 0.001
STR (%)	35.9 ± 3.3	38.8 ± 4.0	0.0293
ER (%)	29.3 ± 2.4	31.0 ± 2.6	0.0536
LVWI (mmHg^∗^l/(min^∗^m^2^))	4.1 ± 1.0	2.9 ± 0.6	0.0003

**Table 4:** Functional parameters of autonomic nervous system.

Parameter	Rowers (*n* = 20)	Reference group (*n* = 20)	*p*
LFnu-RRI (%)	49.6 ± 16.8	53.2 ± 15.4	0.5146
HFnu-RRI (%)	50.3 ± 16.8	46.7 ± 15.4	0.5146
LF-RRI (ms^2^)	2544.9 ± 3126.2	1127.7 ± 1120.5	0.0269
HF-RRI (ms^2^)	2085.0 ± 1568.8	1467.2 ± 2449.5	0.0148
PSD-RRI (ms^2^)	5324.9 ± 4144.4	3141.3 ± 3696.3	0.0248
LF/HF (n/1)	1.0 ± 0.5	1.2 ± 0.6	0.2854

**Table 5:** Functional parameters of cardiovascular system in a horizontal position.

Parameter	Absolute value		*p*	Change		*p*
	Rowers (*n* = 20)	Reference group (*n* = 20)		Rowers (*n* = 20)	Reference group (*n* = 20)	
HR (n/1)	76.7 ± 7.9	74.4 ± 6.6	0.3389	22.5 ± 7.6	14.3 ± 5.3	0.0005
sBP (mmHg)	151.1 ± 16.0	141.8 ± 11.0	0.1225	21.8 ± 11.8	23.5 ± 8.0	0.6119
dBP (mmHg)	108.9 ± 12.9	103.6 ± 10.0	0.1739	31.1 ± 8.6	27.1 ± 6.1	0.0671
mBP (mmHg)	123.1 ± 12.6	116.6 ± 9.1	0.2085	28.3 ± 9.1	25.8 ± 6.4	0.3424
SI (ml/m^2^)	38.4 ± 6.0	35.7 ± 4.2	0.1278	−21.5 ± 8.2	−5.5 ± 5.7	*p* > 0.001
CI (l/min/m^2^)	2.9 ± 0.3	2.6 ± 0.3	0.0288	−0.3 ± 0.4	0.1 ± 0.3	0.0006
TPRI (dyn^∗^s^∗^m^2^/cm^5^)	3367.4 ± 464.5	3538.4 ± 639.6	0.3767	1034.1 ± 453.1	588.1 ± 544.2	0.0128

## Data Availability

The data used to support the findings of this study are available from the corresponding author upon request.
